# Our voice, our choice: a mixed-methods study exploring the nutritious food and beverage item preferences and perspectives of medically tailored grocery clients

**DOI:** 10.3389/fpubh.2026.1884841

**Published:** 2026-07-27

**Authors:** Michael F. Royer, David P. Hott, Diana Juarez, Amee Madura, Melanie Tate, Cesar Lopez, Sofia Portillo, Abby C. King

**Affiliations:** 1 Stanford Prevention Research Center, Stanford University School of Medicine, Palo Alto, CA, United States; 2 Loaves & Fishes Family Kitchen, San Jose, CA, United States; 3 Department of Epidemiology and Population Health, Stanford University School of Medicine, Stanford, CA, United States

**Keywords:** citizen science, disease prevention, food assistance, food insecurity, food is medicine, health promotion, medically tailored groceries, nutrition

## Abstract

Food is Medicine (FIM) strategies are designed to alleviate diet-related chronic disease by increasing access to nutritious foods. In this cross-sectional, mixed-methods study, 13 medically tailored grocery (MTG) clients utilized a mobile app to capture their MTG item preferences and perspectives. Positive MTG item ratings were highest for vegetables, fruits, and protein items. Real-time qualitative narratives revealed preferences for fresh produce and protein items, alongside concerns about packaged produce and unwanted MTG items. FIM strategies may be optimized by engaging clients in the design of MTG services and incorporating their preferences to increase consumption of MTG items.

## Introduction

Diet-related chronic diseases are preventable health conditions (i.e., obesity, type 2 diabetes) that disproportionately impact vulnerable low-income populations and are the leading cause of death in the United States (1). Food is Medicine (FIM) strategies are designed to prevent and alleviate diet-related chronic diseases by improving access to, and consumption of, healthy foods and beverages (2). Medically tailored groceries (MTG) are an FIM service that deliver healthy food and beverage items to individuals who have been referred for FIM services but do not have a sufficient means of transportation to access those services (3). MTG services advance health equity by promoting access to healthy foods that protect against diet-related chronic diseases among vulnerable individuals with low income and food insecurity (4).

Food insecurity is an economic and social condition involving insufficient access to nutritious food (5). Approximately 13.7% of U.S. households (~18,300,000) experienced food insecurity at some point in 2024 (6). Individuals and households experience food insecurity mostly due to financial constraints, but food insecurity can also arise due to a lack of transportation, low food literacy, or limited access to food assistance services (7–9). Food insecurity has been found to increase the risk of numerous diet-related chronic diseases, including obesity, hypertension, type 2 diabetes, heart disease, and cancer (10). FIM strategies, like MTG, aim to increase access to nutritious food, which is crucial for addressing the increased rates of diet-related chronic disease among adults with low income (4), by delivering healthy foods and beverages directly to the households of those experiencing food insecurity.

The purpose of this research was to begin exploring strategies for optimizing FIM impacts by tailoring MTG services to increase consumption, and reduce waste, of foods and beverages that are delivered to food assistance clients. Two primary objectives in this study included (1) identifying MTG clients' healthy food and beverage preferences, and (2) exploring clients' perspectives of the foods and beverages they received in their MTG bags. Given the intention of FIM strategies is to alleviate diet-related chronic disease by increasing access to and consumption of nutritious foods and beverages, it is essential to provide MTG clients with healthy foods and beverages that they want to consume.

## Materials and methods

### Design

A cross-sectional descriptive study using mixed methods (i.e., quantitative, qualitative) was conducted to determine the food and drink item preferences and perspectives of MTG clients. A participatory approach was implemented using the technology-enabled Our Voice citizen science research method (11). Quantitative data were collected online through a survey, in-person using the multi-lingual Discovery Tool app (12), and in-person again using a physical binder containing frequency counts of green (preferred) and red (non-preferred) stickers indicating participant food and beverage item preferences. Qualitative data were collected using the narrative function within the Discovery Tool app. The study protocol was reviewed and approved by the Stanford University Institutional Review Board.

### Setting

This study was conducted in the South Bay region of the San Francisco Bay Area, California. San José, the trademarked “Capital of Silicon Valley,” is the most populated city in the South Bay and the third most populated city in California. Extreme income disparities and food insecurity persist in the Bay Area (13, 14). Moreover, a 2024 report from the California Budget and Policy Center highlighted California as sharing the highest U.S. state-level poverty rates (17.7%) (15).

### Sample

The participant sample for this study included adult MTG clients residing in the South Bay region of the San Francisco Bay Area and receiving food assistance services from Loaves & Fishes Family Kitchen (LFFK), a registered 501(c) (3) non-profit organization located in San Jose, California. Eligible participants were 18 years of age or older, able to speak and understand English or Spanish, residents of the San Francisco Bay Area, and active clients of LFFK.

To be eligible for MTG services at LFFK, an individual must (1) be enrolled in MediCal (California's Medicaid program); (2) have a Santa Clara Family Health Plan; and (3) be referred for MTG services by a physician, case worker, other service provider, or self. The Santa Clara Family Health Plan uses Foodsmart (16), a virtual food-related healthcare platform, to help determine whether patients are eligible for FIM services (e.g., MTG), which FIM service they are eligible to receive, and where to find a provider (e.g., LFFK) to receive the specific FIM services that they need.

### Study procedures

Participants were recruited from a pool of active LFFK clients receiving MTG services. The recruitment process involved both email and in-person invitations to LFFK clients to participate in a study where they would rate MTG food and beverage items (Phase 1), share their perspectives of MTG food and beverage items (Phase 1), and, at a later date, vote on preferred and non-preferred MTG food and beverage items (Phase 2). Interested clients were asked to complete a brief online screening survey to determine their eligibility to join the study. Eligible screener survey respondents were transitioned to a web page containing an electronic informed consent form where they had the opportunity to read about and join the study. Clients were not offered any compensation for participation. All participants completed the informed consent process prior to study participation.

To begin Phase 1 of the study, participants who electronically signed the informed consent proceeded by completing a brief demographics survey. Participants then learned how to engage as “citizen scientists” (e.g., research participants who collect, analyze, and/or report data) by using the Our Voice Discovery Tool mobile app (12) on a LFFK staff member's phone to share their preferences and perspectives of MTG food and beverage items. The app allows participants to take photos and report ratings and narratives of their local environments, specifically the MTGs being delivered to them. The guiding question which participants responded to when using the Discovery Tool app for this study was the following: “Which food and beverage items do you want and not want to receive, and why?.” In the present study, participants unpacked their MTG bags and used the Discovery Tool app to capture a photo of each food and beverage item, rate the individual food and beverage items (positive, neutral, negative), and then provide a text/audio narrative of their food and beverage item perspectives.

Phase 2 of the study involved a 1-month in-person follow-up with participants to again measure their MTG preferences by having them review a binder containing pictures and labels of food and beverage items that were delivered to them in their MTG bags during Phase 1 of the study and then vote on which items they did or did not want in their future MTG bags. This quantitative process allowed for participants to review food/items that they did not receive in their MTG bags during Phase 1 but would like to receive those items in future MTG bags. For Phase 2, the MTG food and beverage items voted on by participants were categorized by food group (fruits, vegetables, grains and legumes, dairy and dairy alternatives, animal protein, plant protein). Participants were asked to place green stickers on food and beverage items that they definitely would like to be included in their future MTG bags and red stickers on food and beverage items that they definitely would not like to be included in their future MTG bags.

### Statistical analysis

Cross-sectional descriptive analyses were used for all study outcomes. Quantitative data from Phase 1 were descriptively analyzed to explore participants' food and beverage item preferences by food group. Frequency counts were determined by totaling the positive, neutral, and negative ratings assigned by participants to food and beverage items. Percentages were then calculated to determine the proportion of participant ratings that were positive, neutral, or negative for each respective food group.

For Phase 2, quantitative data were again descriptively analyzed to further explore participants' specific food and beverage item preferences. Frequency counts were determined by totaling the green stickers (definitely would like) and red stickers (definitely would not like) for each individual pictured item in the food and beverage item photo binder. Percentages were then calculated to determine the proportion of participants that voted for or against each food and beverage item. Phase 2 descriptive statistics were also categorized by food group.

Qualitative data from Phase 1 food and beverage item narratives were similarly organized by food group and subsequently examined with a content analysis (17). Phase 1 food and beverage item ratings informed the categorization of qualitative narratives into either a “positive” or “negative” theme. An explanatory mixed-methods approach was applied that used qualitative data to help explain the frequencies and percentages depicted in the quantitative descriptive data.

## Results

### Sample characteristics

Adult MTG clients (*n* = 13) were sampled for this study (Table 1). The mean participant age was 62.6 years (SD = 14.3), with participant ages ranging from 41 to 85 years old. Three participants did not provide their age. Women (*n* = 10, 76.9%) made up a majority of the participant sample. Participant racial/ethnic groups consisted of Hispanic or Latino (*n* = 5, 38.5%), Asian (*n* = 4, 30.8%), White (*n* = 3, 23.1%), and Other (*n* = 1, 7.7%).

**Table 1:** Participant characteristics.

Characteristics	Descriptives (%)
Sample	*n* = 13
Age (years)	*M* = 62.6, SD = 14.3^*^
Sex	
*Men*	3 (23.1)
Women	10 (76.9)
Race	
*Asian*	4 (30.8)
*Latino*	5 (38.5)
*White*	3 (23.1)
Other	1 (7.7)

^*^M, Mean, SD, Standard Deviation.

#### Phase 1—quantitative

Participants' food and beverage item preferences (Table 2) were categorized by food group, including fruits (*n* = 25), vegetables (*n* = 91), dairy and dairy alternatives (*n* = 12), grains and legumes (*n* = 30), and animal and plant protein (*n* = 33). Fruits received 25 total ratings with 20 positive (80%), 2 neutral (8%), and 3 negative (12%). Vegetables received 91 total ratings with 80 positive (88%), 3 neutral (4%), and 8 negative (8%). Dairy and dairy alternatives received 12 total ratings with 8 positive (66%), 2 neutral (17%), and 2 negative (17%). Grains and legumes received 30 total ratings with 21 positive (70%), 2 neutral (7%), and 7 negative (23%). Animal and plant protein received 33 total ratings with 26 positive (79%), 6 neutral (18%), and 1 negative (3%).

**Table 2:** Phase 1—medically tailored grocery item rating frequencies.

Food group	Rating
*Fruits*	*Positive:* **20** (20/25 = **80%**)
	*Neutral:* **2** (2/25 = **8%**)
	*Negative:* **3** (3/25 = **12%**)
*Vegetables*	*Positive:* **80** (80/91 = **88%**)
	*Neutral:* **3** (3/91 = **4%**)
	*Negative:* **8** (8/91 = **8%**)
*Dairy and dairy alternatives*	*Positive:* **8** (8/12 = **66%**)
	*Neutral:* **2** (2/12 = **17%**)
	*Negative:* **2** (2/12 = **17%**)
*Grains and legumes*	*Positive:* **21** (21/30 = **70%**)
	*Neutral:* **2** (2/30 = **7%**)
	*Negative:* **7** (7/30 = **23%**)
*Animal and plant protein*	*Positive:* **26** (26/33 = **79%**)
	*Neutral:* **6** (6/33 = **18%**)
	*Negative:* **1** (1/33 = **3%**)

#### Phase 1—qualitative

Participants' food and beverage item perspectives were similarly categorized by food group and thematically organized by positive, neutral (when available), or negative rating.

Fruits received 18 total comments derived from 15 positive, 1 neutral, and 2 negative ratings. Examples of positive comments for fruits were, “Fruit cups are good dessert, do give me more,” “Love oranges,” and “Apples are nice and sweet.” The lone neutral comment emphasized, “Would like more of every fruit.” Two negative comments for fruits stated, “I just don't like apple sauce” and “It's OK for me but I like fresh fruit.”

Vegetables received 53 total comments derived from 47 positive, 2 neutral, and 4 negative ratings. Examples of positive comments for vegetables were, “I use onion for all my recipes,” “I love avocados…Would like more,” and “I use a lot of it in salad. This amount is sufficient.” Two neutral comments stated, “I never eat it” and “This amount is enough.” Examples of negative comments were, “Vegetables are not very fresh,” “I prefer fresh green beans or any beans over canned,” and “It causes gas.”

Dairy and dairy alternatives received 5 total comments derived from 8 positive, 2 neutral, and 2 negative ratings. The 2 positive comments for dairy and dairy alternatives stated, “very good, we would like more of it” and “I really liked the milk.” The one neutral comment said, “I only want this once in a while because I never finish it.” The 2 negative comments stated, “I drink very little. It has to be lactose free” and “I don't drink this.”

Grains and legumes received 19 total comments derived from 21 positive, 2 neutral, and 7 negative ratings. Examples of positive comments for grains and legumes were, “This amount is good,” “Cheerios are very convenient in their packaging,” and “I haven't really made brown rice before, but I do like to get new things to try.” The two neutral comments stated, “I do not use this very much” and “I do prefer tortilla.” Examples of negative comments were, “I don't like it,” “I do not use these,” and “I am not eating this…I don't want this item.”

Animal and plant protein received 22 total comments derived from 26 positive, 6 neutral, and 1 negative rating. Examples of positive comments for animal and plant protein were, “I would like to receive a bit more,” “This is very helpful and I love it,” and “I really like the eggs.” Examples of neutral comments were, “I don't eat meat, so tuna is good” and “This is wonderful, however, I don't use turkey meat.” The lone negative comment stated, “I don't like too much tofu.”

#### Phase 2—quantitative

Ten participants (*n* = 10) voted on their food and beverage item preferences (Table 3) during Phase 2 of the study. Food and beverage items were organized by food group. For brevity, “yes” corresponds to a “definitely would like” vote.

**Table 3:** Phase 2—medically tailored grocery item voting outcomes.

Food group	Food Item	Yes	No
*Fruits*	Apple	10/10 (**100%)**	0/10 (**0%**)
	Apple sauce	5/10 (**50%**)	5/10 (**50%**)
	Kiwi	9/10 (**90%**)	1/10 (**10%**)
	Mixed fruit cup	5/10 (**50%**)	5/10 (**50%**)
	Orange	10/10 (**100%**)	0/10 (**0%**)
	Pear	10/10 (**100%**)	0/10 (**0%**)
*Vegetables*	Avocado	10/10 (**100%**)	0/10 (**0%**)
	Celery	5/10 (**50%**)	5/10 (**50%**)
	Cilantro	10/10 (**100%**)	0/10 (**0%**)
	Corn	10/10 (**100%**)	0/10 (**0%**)
	Eggplant	5/10 (**50%**)	5/10 (**50%**)
	Green beans	6/10 (**60%**)	4/10 (**40%**)
	Jalapeño	6/10 (**60%**)	4/10 (**40%**)
	Onion	9/10 (**90%**)	1/10 (**10%**)
	Sweet potato	7/10 (**70%**)	3/10 (**30%**)
	Tomato	8/10 (**80%**)	2/10 (**20%**)
*Dairy and dairy alternatives*	1% Low-fat milk	7/10 (**70%**)	3/10 (**30%**)
	Almond milk	8/10 (**80%**)	2/10 (**20%**)
	Nonfat plain yogurt	8/10 (**80%**)	2/10 (**20%**)
*Grains and legumes*	Beans—black	7/10 (**70%**)	3/10 (**30%**)
	Beans—pinto	6/10 (**60%**)	4/10 (**40%**)
	Bread rolls	6/10 (**60%**)	4/10 (**40%**)
	Cereal	8/10 (**80%**)	2/10 (**20%**)
	Long grain rice	5/10 (**50%**)	5/10 (**50%**)
	Oatmeal	7/10 (**70%**)	3/10 (**30%**)
	Tortillas	6/10 (**60%**)	4/10 (**40%**)
*Animal protein*	Eggs	10/10 (**100%**)	0/10 (**0%**)
	Ground beef	7/10 (**70%**)	3/10 (**30%**)
	Ground turkey	7/10 (**70%**)	3/10 (**30%**)
	Salmon	10/10 (**100%**)	0/10 (**0%**)
	Tuna	9/10 (**90%**)	1/10 (**10%**)
Plant protein	Tofu	5/10 (**50%**)	5/10 (**50%**)

Fruits that were voted most favorably to least favorably included apple (10/10 Yes, 100%), orange (10/10 Yes, 100%), pear (10/10 Yes, 100%), kiwi (9/10 Yes, 90%), apple sauce (5/10 Yes, 50%), and mixed fruit cup (5/10 Yes, 50%).

Vegetables that were voted on included avocado (10/10 Yes, 100%), cilantro (10/10 Yes, 100%), corn (10/10 Yes, 100%), onion (9/10 Yes, 90%), tomato (8/10 yes, 80%), sweet potato (7/10 Yes, 70%), green beans (6/10 Yes, 60%), jalapeño (6/10 Yes, 60%), celery (5/10 yes, 50%), and eggplant (5/10 yes, 50%).

Dairy and dairy alternatives that were voted on included almond milk (8/10 yes, 80%), non-fat plain yogurt (8/10 yes, 80%), and 1% low-fat milk (7/10 yes, 70%).

Grains and legumes that were voted on included cereal (8/10 yes, 80%), black beans (7/10 yes, 70%), oatmeal (7/10 yes, 70%), pinto beans (6/10 yes, 60%), bread rolls (6/10 yes, 60%), tortillas (6/10 yes, 60%), and long grain rice (5/10 yes, 50%).

Animal and plant protein items that were voted on included eggs (10/10 yes, 100%), salmon (10/10 yes, 100%), tuna (9/10 yes, 90%), ground beef (7/10 yes, 70%), ground turkey (7/10 yes, 70%), and tofu (5/10 yes, 50%).

## Discussion

This cross-sectional study used mixed research methods to explore food assistance client preferences and perspectives of MTG food and beverage items. The easy-to-use *Our Voice* Discovery Tool app was utilized to allow clients to capture photos of food and beverage items, rate each item, and provide a narrative that explained each rating. Clients then voted on specific food and beverage items they would like included in their future MTG bags.

Across all food groups, fruits and vegetables had the two highest proportions of positive ratings (i.e., most preferred) among clients. Vegetables received the highest proportion of positive ratings and had over two times more total ratings than any of the other four food groups. Clients shared positive perspectives about fresh, whole fruits and vegetables and negative perspectives on packaged fruits (e.g., apple sauce) and vegetables (e.g., canned green beans). These findings align with past research indicating that over half of food assistance clients preferred fresh fruits and vegetables instead of frozen or canned fruits and vegetables (18). Moreover, several clients expressed their desire to receive more whole fruits and vegetables in future MTG bags. Numerous perspectives for fruits and vegetables included statements from clients that emphasized how they “like” or “love” those healthy food items.

Conversely, dairy and dairy alternatives had the lowest proportion of positive ratings across all food groups and contained the lowest quantity of total ratings. Comments on dairy and dairy alternatives included clients either liking the dairy product (*i.e.*, milk, yogurt) or not using it. Dietary conditions (e.g., lactose intolerance) were also mentioned in one participant's dairy and dairy alternative narrative. A recent study on food insecurity and health inequities pertaining to food allergies/sensitivities produced findings that highlighted how food assistance programs often lack thorough consideration and necessary accommodations for clients with special dietary needs (19). In the present study, perspectives on grains and legumes similarly involved several clients expressing their preference for a different food item (e.g., bread loaves instead of tortillas, brown rice instead of white rice). The discoveries made for both dairy and dairy alternatives and grains and legumes underscore the importance of adequately tailoring MTGs to increase intake and reduce waste of the provided food and beverage items.

This study helps to address a critical gap in the FIM field of research by taking a novel, initial approach to optimizing MTG services. No study to our knowledge has explored the preferences and perspectives of active MTG clients about the food/item contents in their MTG bags. One study used findings from prior research on food assistance client perspectives of foodbank food and beverage items (20) to inform the design of a bi-monthly food box tailored for adults with type 2 diabetes and food insecurity (21). The present study was novel in seeking to optimize MTG services by gathering relevant input directly from food assistance clients actively receiving MTG services.

Gathering MTG client input about their food and beverage item preferences and perspectives yields actionable insights for food assistance organizations to optimize FIM approaches by tailoring food services to clients' personal and cultural needs. FIM approaches are strategically designed to improve health conditions by alleviating diet-related chronic disease among individuals who cannot afford to eat a balanced diet due to low income and food insecurity (22). To ensure MTG services serve their intended purpose of improving health conditions by promoting a healthful diet, MTG bags should contain healthy food and beverage items that clients prefer to consume rather than discard. From FIM approach optimization to maximizing the return-on-investment for FIM services, delivering food and beverage items that are desirable to MTG clients could maximize health improvements and minimize waste.

First, this study made modest contributions toward addressing a gap in the FIM literature concerning MTG clients' nutritious food and beverage preferences. Second, the mixed-methods approach employed in this study produced qualitative MTG item findings that provided meaningful context to the quantitative MTG item preference findings. Third, both English speakers and Spanish speakers were sampled for this study, which allowed for broader inclusion of Hispanic/Latino clients and their culturally relevant food and beverage item preferences and perspectives. Fourth, findings from this small study provide actionable insights for food assistance organizations to gauge MTG clients' healthy food and beverage preferences to promote consumption of MTG items and reduce food waste. The partnering food assistance organization is in the process of actively utilizing data derived from the participants to improve their MTG services.

The present study also had numerous limitations. First, the descriptive nature of this observational study yielded only frequency and percentage statistics for outcomes without further statistical analysis-derived evidence to help explain participants' food and beverage item preferences. Second, a convenience sample of MTG clients was recruited for participation, which meant the participant sample was not adequately representative of MTG clients in the San Francisco Bay Area and the study findings therefore have reduced generalizability. Third, a few food and beverage item preference observations had positive preference ratings accompanied by negatively toned narratives, suggesting that some MTG clients may not have been sufficiently informed and/or did not adequately understand some of the study expectations. However, such mismatches were rare and represented only three percent of all Phase 1 observations. Fourth, the small sample of participants recruited for this study limited the reliability, namely the precision, of the study findings due to a relatively low number of observed healthy food and beverage item preferences and perspectives. Thus, a larger sample of participants is needed to establish a more complete understanding of healthy food and beverage item preferences among MTG clients in this area.

## Conclusion

This study was novel in utilizing mixed-methods research to explore the healthy food and beverage item preferences and perspectives of MTG clients. Overall, a high proportion of this small sample of MTG clients preferred whole fruits and vegetables along with protein-rich food items, while several clients shared perspectives expressing their preference for different dairy and dairy alternative items and grains and legumes items. This study represented a modest yet meaningful step toward optimizing FIM strategies to maximize MTG item intake and reduce food waste. Future studies should recruit a larger, more representative sample of MTG clients and test associations between MTG clients' healthy food and beverage item preferences and their relevant personal, behavioral, and health-related outcomes.

## Data Availability

The raw data supporting the conclusions of this article will be made available by the authors, without undue reservation.
